# Pharmacological Characterization of 5-HT_1A_ Autoreceptor-Coupled GIRK Channels in Rat Dorsal Raphe 5-HT Neurons

**DOI:** 10.1371/journal.pone.0140369

**Published:** 2015-10-13

**Authors:** Alberto Montalbano, Renato Corradetti, Boris Mlinar

**Affiliations:** Department of Neuroscience, Psychology, Drug Research and Child Health, University of Florence, Florence, Italy; Dalhousie University, CANADA

## Abstract

G protein-activated inwardly rectifying potassium (GIRK) channels in 5-HT neurons are assumed to be principal effectors of 5-hydroxytryptamine 1A (5-HT_1A_) autoreceptors, but their pharmacology, subunit composition and the role in regulation of 5-HT neuron activity have not been fully elucidated. We sought for a pharmacological tool for assessing the functional role of GIRK channels in 5-HT neurons by characterizing the effects of drugs known to block GIRK channels in the submicromolar range of concentrations. Whole-cell voltage-clamp recording in brainstem slices were used to determine concentration-response relationships for the selected GIRK channel blockers on 5-HT_1A_ autoreceptor-activated inwardly rectifying K^+^ conductance in rat dorsal raphe 5-HT neurons. 5-HT_1A_ autoreceptor-activated GIRK conductance was completely blocked by the nonselective inwardly rectifying potassium channels blocker Ba^2+^ (EC_50_ = 9.4 μM, full block with 100 μM) and by SCH23390 (EC_50_ = 1.95 μM, full block with 30 μM). GIRK-specific blocker tertiapin-Q blocked 5-HT_1A_ autoreceptor-activated GIRK conductance with high potency (EC_50_ = 33.6 nM), but incompletely, i.e. ~16% of total conductance resulted to be tertiapin-Q-resistant. U73343 and SCH28080, reported to block GIRK channels with submicromolar EC_50_s, were essentially ineffective in 5-HT neurons. Our data show that inwardly rectifying K^+^ channels coupled to 5-HT_1A_ autoreceptors display pharmacological properties generally expected for neuronal GIRK channels, but different from GIRK1-GIRK2 heteromers, the predominant form of brain GIRK channels. Distinct pharmacological properties of GIRK channels in 5-HT neurons should be explored for the development of new therapeutic agents for mood disorders.

## Introduction

It is well documented that the activity of raphe 5-HT neurons is under regulatory control by 5-HT_1A_ autoreceptors and K^+^ channels. Early electrophysiological studies *in vivo* [1] and in midbrain slices [2] suggested that stimulation of 5-HT receptors hyperpolarize dorsal raphe 5-HT neurons by an increase in K^+^ conductance. Using intracellular recordings, Williams *et al*. [3] showed that stimulation of 5-HT or GABA_B_ receptors on dorsal raphe 5-HT neurons activates an inwardly rectifying K^+^ conductance via a pertussis toxin sensitive G-protein. This conductance was abolished by low (100 μM) concentration of Ba^2+^. Studies using whole-cell [4] and single-channel [5] patch-clamp recording on acutely isolated dorsal raphe 5-HT neurons confirmed the sensitivity to pertussis toxin and the block by Ba^2+^. These studies further revealed that the receptor implicated belongs to the 5-HT_1A_ subtype and that the activation of K^+^ channels occurs via a direct membrane-limited pathway without the involvement of soluble intracellular messengers. Similar findings were reported by Bayliss *et al*. [6] for 5-HT neurons in the medulla oblongata (raphe pallidus and raphe obscurus). Together, these studies clearly indicate that the inwardly rectifying K^+^ channel activated by 5-HT_1A_ autoreceptors belongs to the GIRK (K_ir_3) channel family. Consistently, it has been shown that GIRK1 (K_ir_3.1), GIRK2 (K_ir_3.2) and GIRK3 (K_ir_3.3) mRNA and proteins are expressed in dorsal raphe 5-HT neurons [7–10].

In raphe 5-HT neurons, the activation of 5-HT_1A_ autoreceptors, besides the opening of GIRK channels, also produces an inhibition of voltage-gated Ca^2+^ channels [11–13] and, probably, modulate other ion channel types [3,4]. The precise role of GIRK channels in the control of 5-HT neuron activity has remained unclear, in part due to the limited knowledge of their pharmacological properties. Ba^2+^, a nonselective blocker of inwardly rectifying K^+^ channels, is the only drug so far reported to block GIRK channels in raphe 5-HT neurons, albeit with unknown EC_50_. Given the functionally important role of 5-HT_1A_ autoreceptor-activated inwardly rectifying K^+^ channels in the control of 5-HT neuron activity, the availability of organic blockers would greately help further functional studies and may lead to the development of valuable new therapeutic agents. Here, we used whole-cell recording in brainstem slices to characterize the effects of available GIRK channel blockers on inwardly rectifying K^+^ current activated by 5-HT_1A_ autoreceptors in 5-HT neurons. We found that inwardly rectifying K^+^ channels coupled to 5-HT_1A_ autoreceptors have pharmacological properties generally expected from GIRK channels, although the profile of blocker sensitivity differs from that of GIRK1-GIRK2 heteromers, the predominant form of brain GIRK channels.

## Materials and Methods

### Animal Welfare and Ethical Statement

All animal care and experimental procedures complied with the European Communities Council Directive (2010/63/UE) and were approved by the Internal Committee for Animal Care and Experimental Use (IACUC) of the University of Florence and communicated to the Italian Ministry of Health, as required (D.L. 116/92). A total of 22 male Wistar rats (Harlan Italy, Milano, Italy) were used in the present study. Animals were housed under standard laboratory conditions (12 h light/dark cycle, ambient temperature 22±1°C, humidity 40–50%, standard chow and water *ad libitum*). Animals (25–35 days of age at the experimental day) were sacrified under isoflurane anesthesia, and all efforts were made to minimize suffering.

### Whole-cell patch-clamp recordings

Experimental procedures have been previously described in detail [14]. In brief, the brain was rapidly removed and dissected in ice-cold gassed (95% O_2_ and 5% CO_2_) artificial cerebrospinal fluid (ACSF) composed of: 124 mM NaCl, 2.75 mM KCl, 1.25 mM NaH_2_PO_4_, 1.3 mM MgCl_2_, 2 mM CaCl_2_, 26 mM NaHCO_3_, 11 mM D-glucose. The brainstem was sliced coronally into 200 μm thick slices with a vibratome (DSK, T1000, Dosaka, Japan). After recovery for at least 90 min at room temperature, the slices were individually transferred to the recording chamber and superfused continuously with warmed (29–31°C; Warner Instruments in-line heater TC324-C), modified (see below) ACSF at a rate of 2 mL min^-1^. Slices were allowed to equilibrate for at least 15 min before the beginning of the recording. Drugs were bath-applied through a peristaltic pump-driven perfusion system and a complete exchange of the recording chamber volume occurred in approximately 1 min. Neurons within the dorsal raphe nucleus were visualized by infrared differential interference contrast (IR-DIC) video microscopy with a Newicon camera (C2400-07; Hamamatsu, Hamamatsu City, Japan) mounted on an upright microscope (Axioskop; Zeiss, Göttingen, Germany). Recordings were made using an EPC-10 amplifier (HEKA Elektronic, Lamberecht, Germany). Patch pipettes were prepared from thick-walled borosilicate glass on a P-97 Brown-Flaming electrode puller (Sutter Instruments, Novato, CA, USA). The pipette solution consisted of: 120 mM K gluconate, 15 mM KCl, 2 mM MgCl_2_, 10 mM HEPES, 0.1 mM EGTA, 10 mM Na_2_phosphocreatine, 4 mM MgATP, 0.3 mM Na_3_GTP (pH 7.35 with ≈ 9 mM KOH). Pipettes had filled-tip resistance of 2.5–5.2 MΩ.

To block synaptic transmission, all recordings were done using modified ACSF supplemented with a cocktail of glutamate and GABA/glycine receptor blockers consisting of: 10 μM NBQX (2,3-dioxo-6-nitro-1,2,3,4-tetrahydrobenzo[f]quinoxaline-7-sulfonamide disodium salt), 20 μM D-AP5 (D-(-)-2-amino-5-phosphonopentanoic acid), 10 mM strychnine hydrochloride, 10 μM SR-95531 (6-imino-3-(4-methoxyphenyl)-1(6H)-pyridazinebutanoic acid hydrobromide) and 2 μM CGP-55845 (3-N[1-(S)-(3,4-dichlorophenyl)ethyl]amino-2-(S)-hydroxypropyl-P-benzyl-phosphinic acid hydrochloride). The modified ACSF also contained 5.5 mM K^+^ (the additional 2.75 mM by Na^+^ substitution) to increase the driving force for inward K^+^ current and to shift K^+^ reversal potential to a more positive value, thus permitting reliable detection of inwardly rectifying K^+^ currents at membrane potentials more negative than -85 mV, at which there is a neglibile contribution of outward rectifying K+ currents and voltage-gated ion channels. We used hyperpolarizing voltage ramps from the holding potential of -65 mV (to -125 mV, every 10 s; 100 mV s^-1^; 3 kHz cutoff frequency low-pass filter; 10 kHz sampling frequency) and measured the conductance from the slope of inward K^+^ current in range from -110 to -90 mV (G_-110/-90 mV_). To monitor access resistance throughout the recording, hyperpolarizing pulses (10 mV; 100 ms duration; 16 kHz low-pass filter; 25 kHz sampling frequency; cell capacitance cancellation circuit switched off) were interlaced with ramps. The access resistance was in 8 to 25 MΩ range and recordings were interrupted when it changed for more than 25%. In addition, approximately 15% of the experiments were aborted since they were compromised by spontaneous activation of a marked outward rectifying K^+^ current, similar to that previously described by Bayliss *et al*. [6], which was not further examined. The membrane potential was not corrected for Donnan liquid junction potential. The effects of channel blockers were examined using a cumulative concentration-response protocol in the contunuous presence of the 5-HT_1A_ receptor agonist 5-carboxamidotryptamine maleate (5-CT). One experiment was performed per slice. Each concentration of blocker was bath-applied until the apparent steady-state of the online monitored G_-110/-90_ values was reached, i.e. typically eight to ten minutes, with the exception of SCH23390 (up to 20 min). In most of recordings three to five increasing concentrations of a blocker were succesfully applied and the concentration-response relationship in individual neurons could be fitted by the four parameters logistic equation y = b+ (a—b)/(1 + (EC_50_ /[Blocker])^nH^), where *a* corresponds to G_-110/-90_ in the absence of blocker effect, *b* corresponds to G_-110/-90_ with the maximal blocker effect, EC_50_ is the half-maximally effective concentration and n_H_ is the Hill coefficient.

### Materials

Stock solutions of 5-CT, BaCl_2_, tertiapin-Q, SCH23390 [(R)-(+)-7-Chloro-8-hydroxy-3-methyl-1-phenyl-2,3,4,5-tetrahydro-1H-3-benzazepine hydrochloride] were prepared in water and those of SCH28080 (2-Methyl-8-(phenylmethoxy)imidazo[1,2-a]pyridine-3-acetonitrile) and U73343 (1-[6-[[(17β)-3-Methoxyestra-1,3,5(10)-trien-17-yl]amino]hexyl]-2,5-pyrrolidinedione) in DMSO. All stock solutions, which were at least a thousand times the highest experimental concentration, were aliquoted and stored at -20°C until use. The highest experimental concentration of DMSO was 0.05%. 5-CT, SCH23390 and U73343 were purchased from Tocris (Tocris Bioscience, Bristol, UK); SCH28080 from HelloBio (Bristol, UK); CGP-55845; D-AP5, SR-95531, NBQX from Abcam (Cambridge, U.K.); tertiapin-Q from Abcam and Tocris; Isoflurane from Baxter S.p.A. (Rome, Italy); HEPES, ATP and DMSO from Fluka (St. Gallen, Switzerland). All other substances were obtained from Sigma-Aldrich (Milano, Italy)

### Data analysis and statistical procedures

Data were analyzed using Patchmaster 2 (HEKA Elektronic) and then with Prism 6 software (GraphPad Software, San Diego, CA, USA). All statistics are given as mean ± SD, except EC_50_ values which are given as mean and 95% confidence intervals (95% C.I.).

## Results

**Fig 1:**
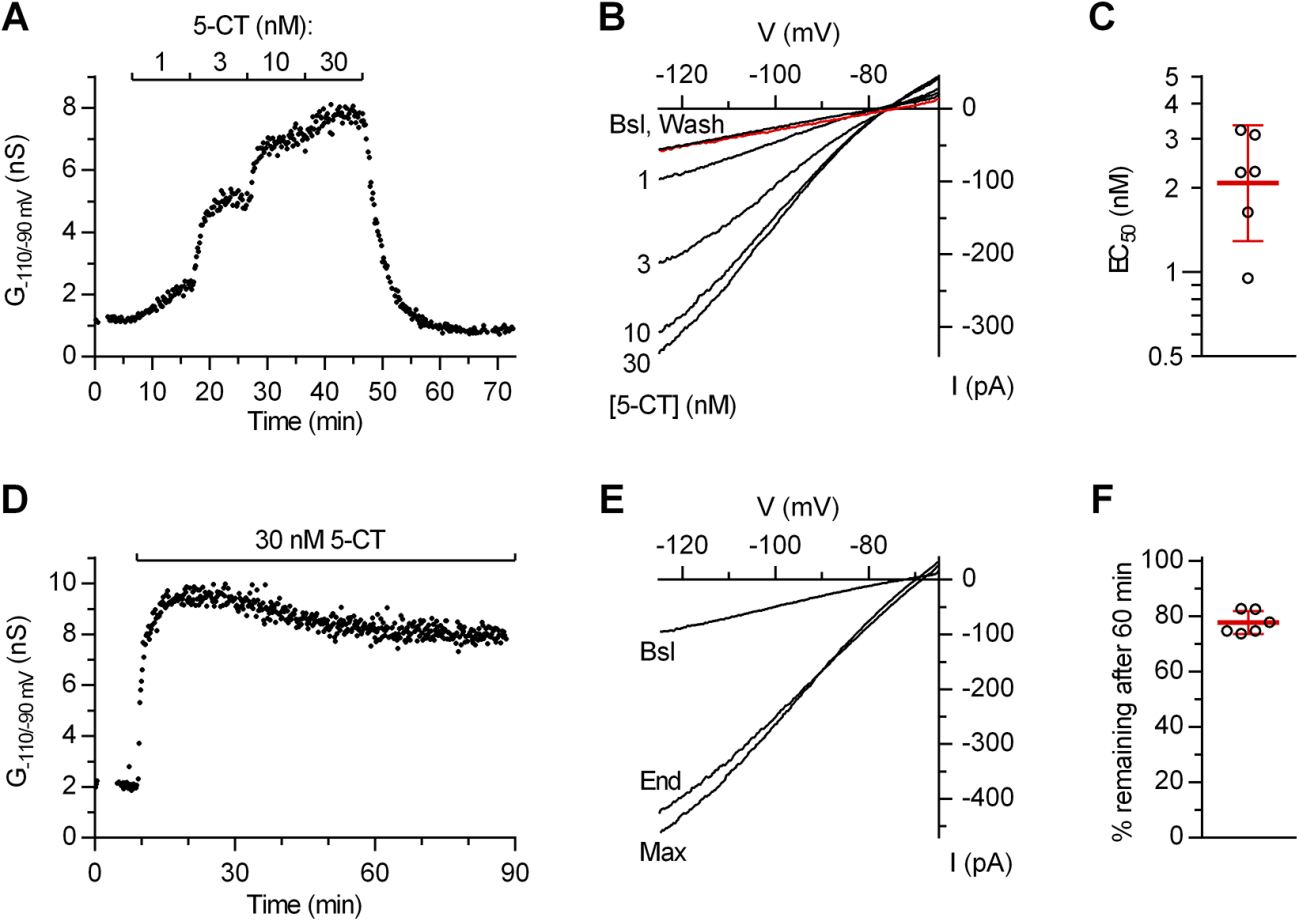
5-CT-activated inwardly rectifying K^+^ conductance in 5-HT neurons displays limited desensitization. (A) Time-course of a representative experiment (*n* = 6) showing the effect of increasing concentrations of bath applied 5-HT_1A_ receptor agonist, 5-CT on inwardly rectifying K^+^ conductance (G_-110/-90 mV_) in a dorsal raphe 5-HT neuron. Extracellular solution contained 5.5 mM K^+^ and a mix of synaptic blockers (see methods). In this and the following Figs, time indicates duration of whole-cell configuration. (B) Current-voltage plot of the same experiment. Traces are averages of the last 13 individual ramps recorded before 5-CT application (Bsl); at the indicated 5-CT concentrations and following the washout of 5-CT (Wash; red trace). (C) Scatter plot of EC_50_ values of 5-CT in individual neurons. Bars correspond to geometric mean ± 95% C.I. (D) Time-course of a representative experiment (*n* = 6) showing persistent activation of G_-110/-90 mV_ by 30 nM 5-CT. (E) Current-voltage plot of the same experiment. Traces are averages of 13 consecutive ramps recorded before 5-CT application (Bsl); during the maximal effect (Max) and at the end of the recording (End). (F) Scatter plot showing the percentage of maximal 5-CT effect (≈10 min in 5-CT) remaining 60 min after reaching the maximum (≈70 min in 5-CT) in individual recordings. Bars correspond to mean ± SD.

To activate 5-HT_1A_ autoreceptors in dorsal raphe 5-HT neurons, we used 5-CT, an agonist which in our experimental conditions selectively activates 5-HT_1A_ autoreceptors [15,14] and is structurally similar to the endogenous agonist, 5-HT. As shown in Fig 1A–1C, bath application of 5-CT produced a concentration-dependent increase in an inwardly rectifying K^+^ conductance which was completely reversed upon agonist washout. To minimize 5-HT_1A_ receptor and GIRK channel desensitization we used 5-CT at 30 nM, a concentration which produces near-maximal effect. As shown in Fig 1D–1F, at this concentration only a limited run-down of the response was observed over a prolonged period of continuous agonist application (22.3 ± 4.1% in 1 h, mean ± SD, *n* = 6), permitting a fairly accurate determination of concentration-response relationships for the investigated blockers in individual neurons.

**Fig 2:**
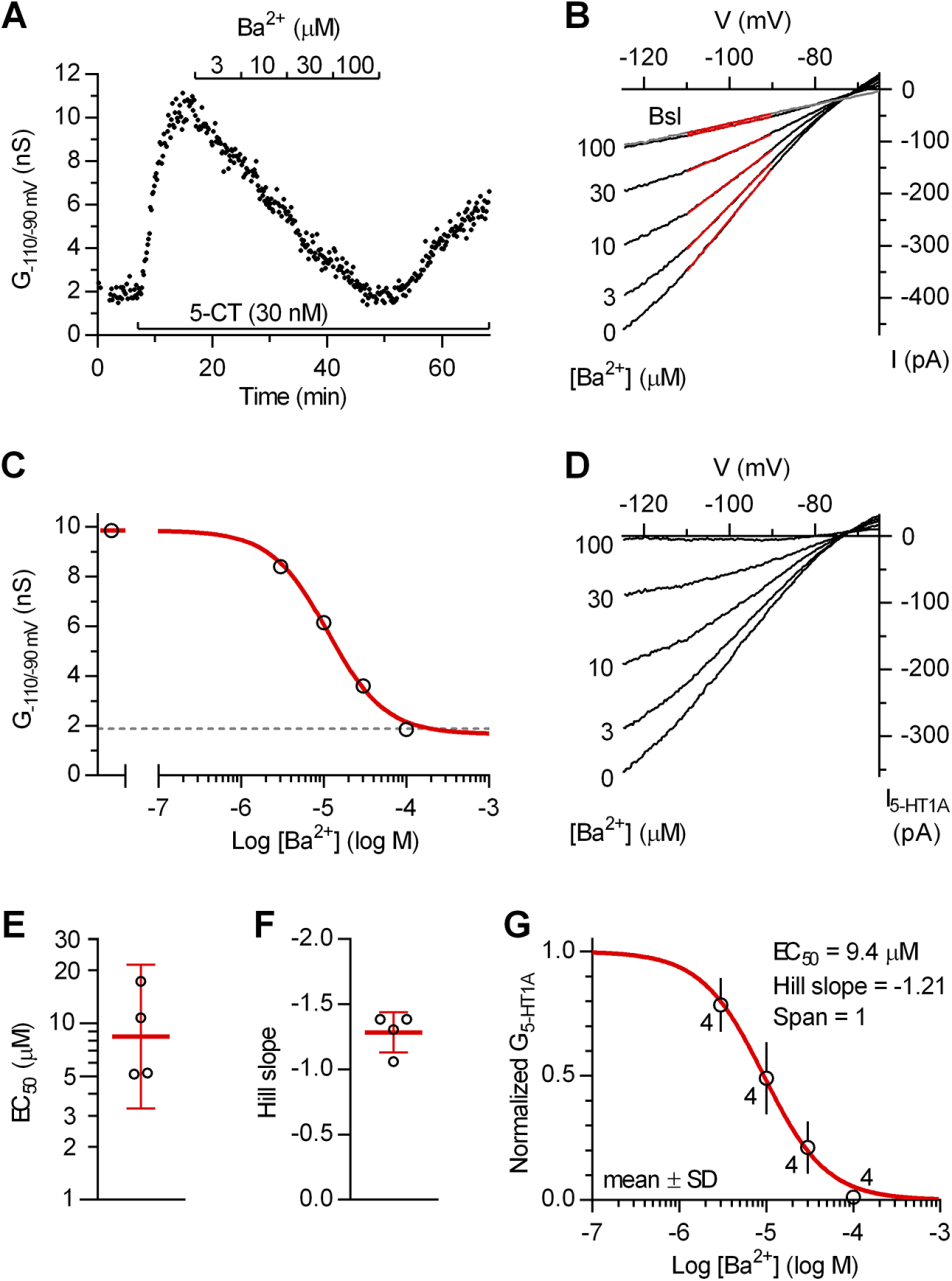
Block of 5-HT_1A_ autoreceptor-activated inwardly rectifying K^+^ conductance by Ba^2+^. (A-D) Representative experiment. (A) Time-course of a representative experiment (*n* = 4) illustrating the concentration-dependent block of 5-CT-induced inwardly rectifying K^+^ conductance (G_-110/-90 mV_) by Ba^2+^. (B) Current-voltage plot of the same experiment. Traces are averages of the last 7 individual ramps recorded before 5-CT application (Bsl; grey trace) and at the indicated concentrations of Ba^2+^. Red lines represent linear fits of data from -110 to -90 mV used to compute G_-110/-90 mV_. (C) Graph illustrating calculation of Ba^2+^ concentration-response curve in individual recordings. Symbols correspond to the slope of the respective red lines shown in B. Dashed line represents the baseline slope. The red curve is the data fit with the function y = b + (a - b)/(1 + (EC_50_ /[Ba^2+^])^nH^), where *a* corresponds to the slope conductance in the absence of Ba^2+^, *b* corresponds to the maximal Ba^2+^ effect, EC_50_ is the half-maximally effective Ba^2+^ concentration and n_H_ is the Hill coefficient. (D) Current-voltage plot of net 5-CT-induced current (I_5-HT1A_) obtained by subtraction of the baseline current (gray trace in B). (E) Scatter plot of EC_50_ values of Ba^2+^ in individual neurons. Bars correspond to geometric mean ± 95% C.I. (F) Scatter plot of Hill slope values of Ba^2+^ in individual recordings. Bars correspond to mean ± SD. (G) Average concentration-response for Ba^2+^ on normalized data from all experiments. Data are normalized from zero (Bsl) to one (5-CT with zero Ba^2+^) and thus correspond to net 5-HT_1A_ receptor-activated inwardly rectifying K^+^ conductance (G_5-HT1A_). Red line is the best least-squares fit to the logistic equation, y = 1/(1 + (EC_50_ /[Ba^2+^])^nH^), where EC_50_ is the half-maximally effective concentration and n_H_ is the Hill coefficient (R^2^ = 0.894).

We first wanted to characterize the antagonism of 5-HT_1A_ receptor-activated inwardly rectifying K^+^ conductance by Ba^2+^, an information surprisingly missing even if Ba^2+^ is the only blocker so far reported to be effective in 5-HT neurons. As shown in Fig 2, Ba^2+^ concentration-dependently blocked 5-CT-induced inwardly rectifying K^+^ conductance, producing full block at 100 μM concentration. The full block by Ba^2+^ was confirmed in additional recordings in which 5-CT (30 nM, 10 min) failed to induce inwardly rectifying K^+^ conductance when co-applied in the presence of 100–150 μM Ba^2+^ (10 min; *n* = 5, not shown). In four individual neurons in which Ba^2+^ was applied in concentrations from 3 to 100 μM, fit of data with a logistic equation revealed EC_50_ values ranging from 5.1 to 17.3 nM and Hill slope values ranging from -1.06 to -1.39 (Fig 2E and 2F). Concentration-response relationship of Ba^2+^ on pooled data (Fig 2G) revealed an EC_50_ of 9.4 μM (95% C.I. 7.2 to 12.2 μM) and a Hill Slope of -1.21 (95% C.I. -0.83 to -1.59).

**Fig 3:**
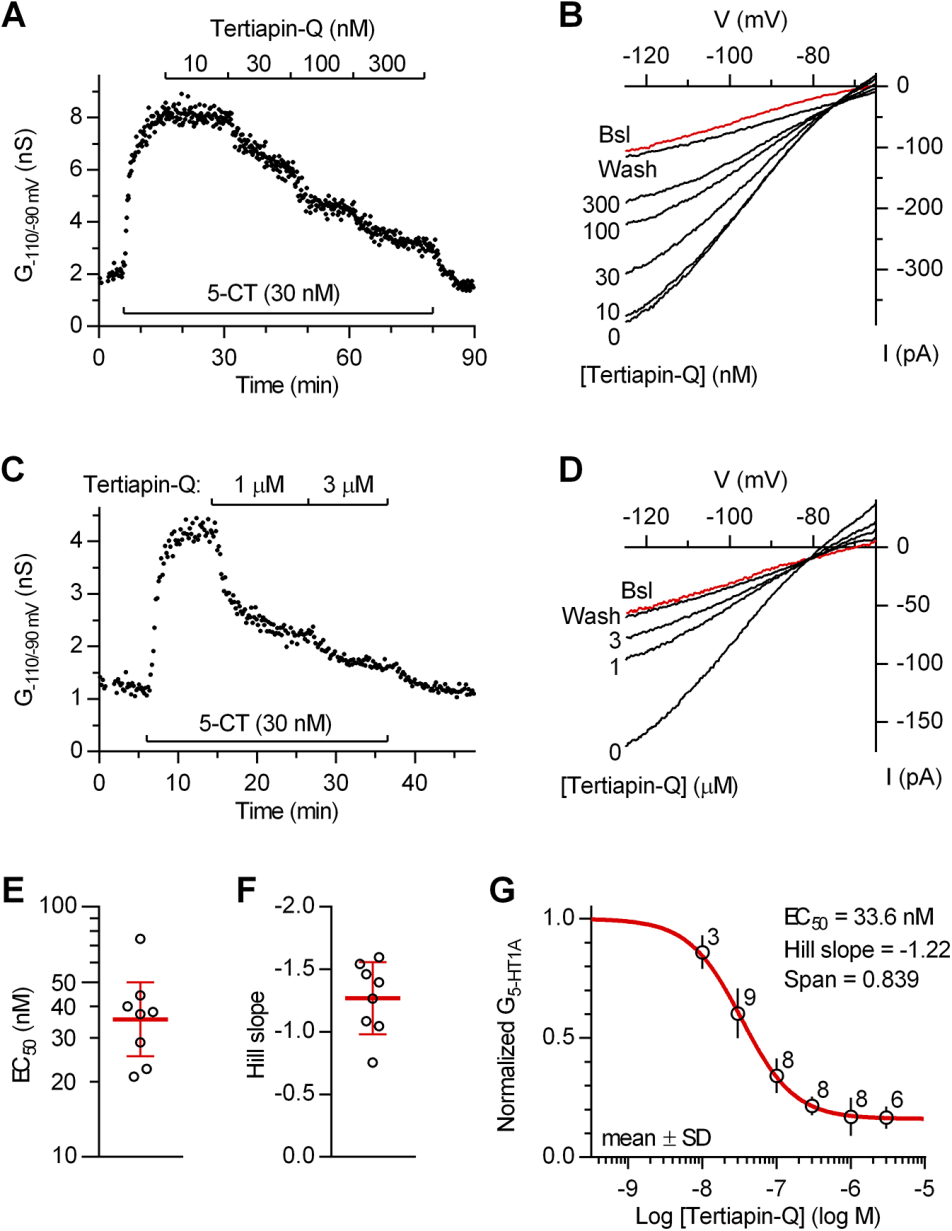
Block of 5-HT_1A_ autoreceptor-activated inwardly rectifying K^+^ conductance by tertiapin-Q. (A) Time-course of a representative experiment (*n* = 8) illustrating concentration-dependent block of 5-CT-induced inwardly rectifying K^+^ conductance (G_-110/-90 mV_) by tertiapin-Q. (B) Current-voltage plot of the same experiment. Traces are averages of the last 7 individual ramps recorded before 5-CT application (Bsl; red trace), at the indicated concentrations of tertiapin-Q, and following the washout of 5-CT and tertiapin-Q (Wash). (C) Time-course of a representative experiment (*n* = 7) illustrating incomplete block of 5-CT-induced inwardly rectifying K^+^ conductance by high concentrations of tertiapin-Q. (D) Current-voltage plot of the same experiment. Traces are averages of the last 11 individual ramps recorded before 5-CT application (Bsl; red trace), at the indicated concentrations of tertiapin-Q, and following the washout of 5-CT and tertiapin-Q (Wash). (E) Scatter plot of EC_50_ values of tertiapin-Q in individual neurons. Bars correspond to geometric mean ± 95% C.I. (F) Scatter plot of Hill slope values of tertiapin-Q in individual recordings. Bars correspond to mean ± SD. (G) Average concentration-response for tertiapin-Q on normalized data corresponding to net 5-HT_1A_ receptor-activated inwardly rectifying K^+^ conductance (G_5-HT1A_) from all experiments. Red line is the best least-squares fit to the logistic equation, y = b + (1 - b)/(1 + (EC_50_ /[Tertiapin-Q])^nH^), where EC_50_ is the half-maximally effective concentration, n_H_ is the Hill coefficient, and b is the fraction remaining at the maximal tertiapin-Q effect (R^2^ = 0.906).

We next characterized the block of 5-HT_1A_ receptor-activated inwardly rectifying K^+^ conductance by the specific GIRK blocker tertiapin-Q, a non-air-oxidizable derivative of honey bee toxin tertiapin [16]. Tertiapin-Q concentration-dependently blocked 5-CT-induced inwardly rectifying K^+^ conductance in the submicromolar range, but the block was incomplete (e.g. Fig 3A and 3B). To better examine if tertiapin-Q can produce a complete block, in an additional set of experiments, tertiapin-Q was applied only at concentrations of 1 μM (*n* = 2) and/or 3 μM (*n* = 5; e.g. Fig 3C and 3D). In all cases, tertiapin-Q failed to produce full block. In eight individual neurons in which tertiapin-Q was applied at concentrations from 10 or 30 nM to 0.3 or 1 μM, fit of data with a logistic equation showed that tertiapin-Q blocked 5-CT-induced inwardly rectifying K^+^ conductance with EC_50_ values ranging from 20.9 to 74.6 nM and Hill slope values ranging from -0.76 to -1.60 (Fig 3E and 3F). Concentration-response of tertiapin-Q on pooled data from all fifteen experiments (Fig 3G) revealed an EC_50_ of 33.6 nM (95% C.I. 27.8 to 40.7 nM), a Hill Slope of 1.22 (95% C.I. -0.90 to -1.54) and a maximal block of 83.9% (95% C.I. 79.6 to 88.3%).

**Fig 4:**
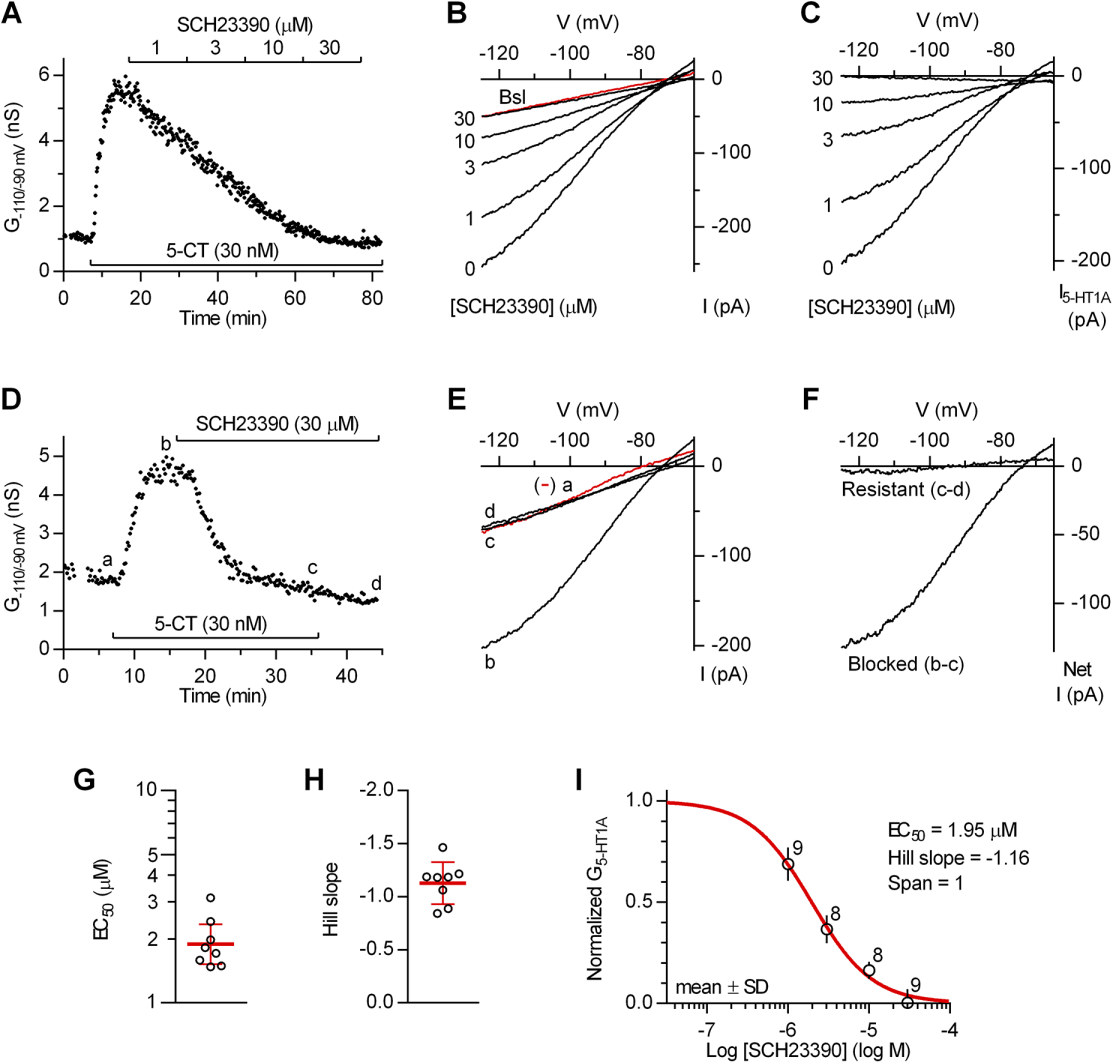
Block of 5-HT_1A_ autoreceptor-activated inwardly rectifying K^+^ conductance by SCH23390. (A) Time-course of a representative experiment (*n* = 8) illustrating concentration-dependent block of 5-CT-induced inwardly rectifying K^+^ conductance (G_-110/-90 mV_) by SCH23390. (B) Current-voltage plot of the same experiment. Traces are averages of the last 7 individual ramps recorded before 5-CT application (Bsl; red trace) and at the indicated concentrations of SCH23390. (C) Current-voltage plot of net 5-CT-induced current (I_5-HT1A_) obtained by subtraction of the baseline current (red trace in B). (D) Time-course of a representative experiment (*n* = 3) illustrating near complete block of 5-CT-induced inwardly rectifying K^+^ conductance by 30 μM SCH23390. (E) The current-voltage plot of the same experiment. Traces are averages of 11 individual ramps recorded before (a; red trace) and after (b) 5-CT application, after the application of SCH23390 (c) and following the washout of 5-CT (d). (F) Current-voltage plot of net SCH23390-blocked and SCH23390-resistant current of the same experiment. (G) Scatter plot of EC_50_ values of SCH23390 in individual recordings. Bars correspond to geometric mean ± 95% C.I. (F) Scatter plot of Hill slope values of SCH23390 in individual recordings. Bars correspond to mean ± SD. (G) Average concentration-response for SCH23390 on normalized data corresponding to net 5-HT_1A_ receptor-activated inwardly rectifying K^+^ conductance (G_5-HT1A_) from all experiments. Red line is the best least-squares fit to the logistic equation, y = 1/(1 + (EC_50_ /[SCH23390])^nH^), where EC_50_ is the half-maximally effective concentration and n_H_ is the Hill coefficient (R^2^ = 0.936).

We proceeded to examine the block of 5-HT_1A_ receptor-activated inwardly rectifying K^+^ conductance by organic compounds reported to block GIRK with EC_50_ values in the submicromolar range. We first tested SCH23390, a classic dopamine D_1_ receptor antagonist and potent 5-HT_2C_ receptor agonist [17] which is also direct GIRK channel blocker [18]. As shown in Fig 4A–4C, SCH23390 concentration-dependently blocked 5-CT-induced inwardly rectifying K^+^ conductance, producing a complete block at 30 μM concentration. The ability of SCH23390 to fully block 5-CT-induced inwardly rectifying K^+^ conductance was confirmed in additional recordings in which it was applied only at 30 μM (*n* = 3; Fig 4D–4F) and in recordings in which 5-CT (30 nM, 10 min) failed to induce inwardly rectifying K^+^ conductance when co-applied in the presence of 30 μM SCH23390 (20 min; *n* = 3, not shown). In eight individual neurons in which SCH23390 was cumulatively applied at concentrations ranging from 1 to 10 or 30 μM, fit of data with logistic equation revealed a block of 5-CT-induced inwardly rectifying K^+^ conductance with EC_50_ values ranging from 1.5 to 3.1 μM and Hill slope values ranging from -0.84 to 1.47 (Fig 4G and 4H). Concentration-response of SCH-23390 on pooled data from all experiments (Fig 4I) revealed an EC_50_ of 1.95 μM (95% C.I. 1.7 to 2.2 μM) and a Hill Slope of -1.16 (95% C.I. -0.98 to -1.35).

**Fig 5:**
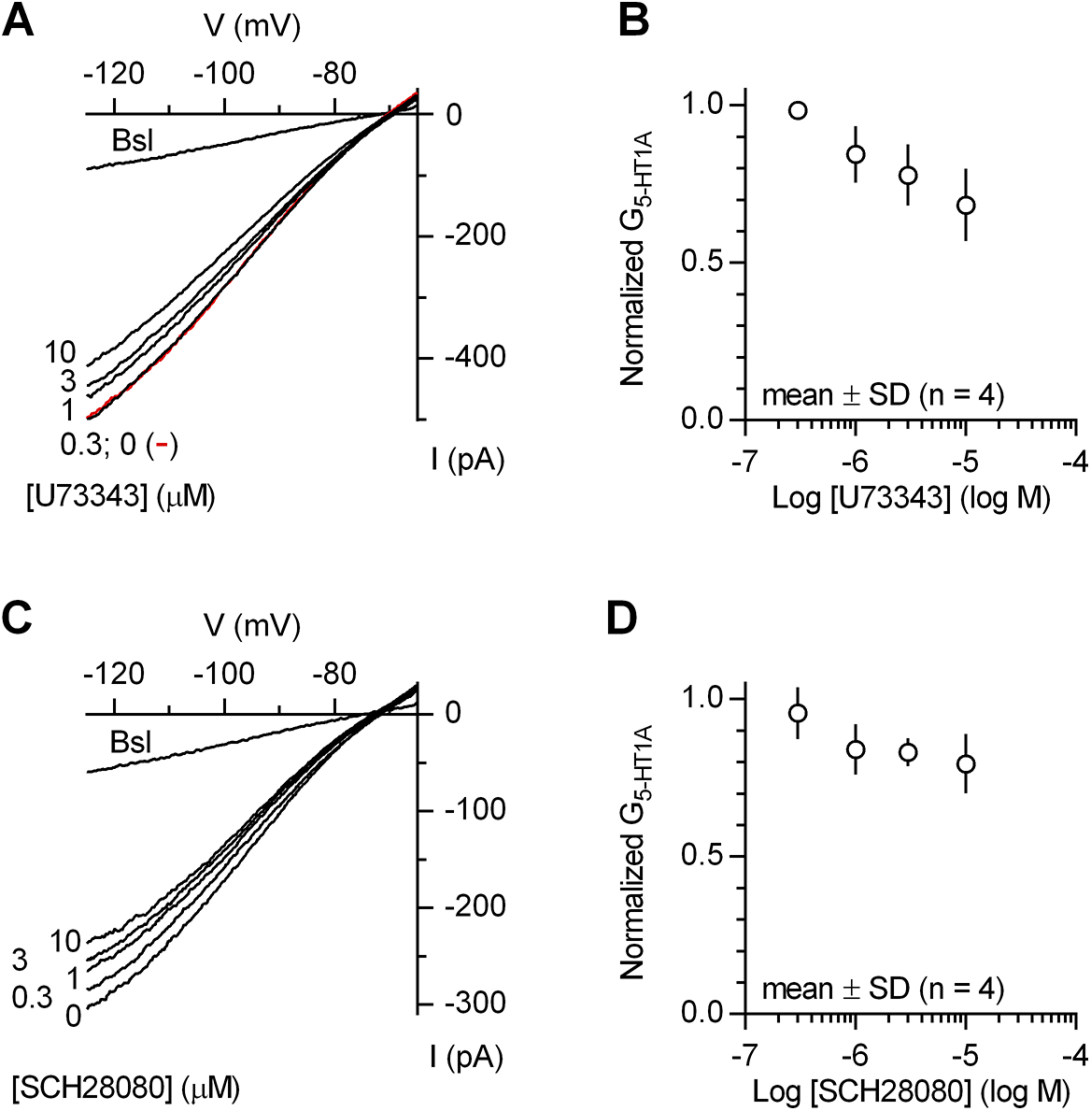
U73343 and SCH28080 are relatively ineffective blockers of 5-HT_1A_ autoreceptor-coupled inwardly rectifying K^+^ channels. (A) Current-voltage plot of a representative experiment (*n* = 4) illustrating weak block of 5-CT-induced inwardly rectifying K^+^ conductance by increasing concentrations of U73343. Each concentration was applied for 10 min. Traces are averages of the last 7 individual ramps recorded before (Bsl) and after 5-CT application (0; red trace), and at the indicated concentrations of U73343. (B) Average concentration-response for U73343 on normalized data corresponding to net 5-HT_1A_ receptor-activated inwardly rectifying K^+^ conductance (G_5-HT1A_) from all experiments. (C) Current-voltage plot of a representative experiment (*n* = 4) illustrating the effect of increasing concentrations of SCH28080 on 5-CT-induced inwardly rectifying K^+^ conductance. Each concentration was applied for 10 min. Traces are averages of the last 7 individual ramps recorded before 5-CT application (Bsl) and at the indicated concentrations of SCH28080. (D) Average concentration-response for SCH28080 on normalized data from all experiments.

We also examined the effect of two small molecules reported to block GIRK channels with EC_50_ values in the submicromolar range: U73343, which blocks GIRK channels in acutely isolated rat neocortical pyramidal cells with an EC_50_ of 400 nM [19], and SCH28080, reported to block mouse GIRK channels in AtT20 and HL-1 cells with EC_50_ values of 200 and 300 nM, respectively [20]. As shown in Fig 5, both substances were poorly effective blockers of the 5-CT-induced inwardly rectifying K^+^ conductance in the dorsal raphe 5-HT neurons. At 10 μM, the highest concentration tested, U73343 and SCH28080 reduced 5-CT-induced inwardly rectifying K^+^conductance by 31.6% (range 22.4–48.0%, *n* = 4) and 20.5% (range 12.3–32.3%, *n* = 4), respectively.

## Discussion

Pharmacological properties of GIRK channels in 5-HT neurons have remained uncharacterized in spite of the evidence that GIRK channels are principal effectors of 5-HT_1A_ autoreceptors. We attempted to find a blocker which may serve as a tool to study the role of GIRK channels in the regulation of 5-HT neuron activity. Whole-cell recording in brainstem slices were used to directly measure GIRK conductance.

We report the first quantitative assessment of the effects of a series of specific organic blockers and Ba^2+^ on native inwardly rectifying K^+^ channels activated by 5-HT_1A_ autoreceptor stimulation in raphe 5-HT neurons.

### Methodological considerations

Pharmacology of neuronal GIRK channels is largely unexplored mostly because of the paucity of GIRK blockers available. In many neuronal types, e.g. pyramidal and Purkinje neurons, studies of GIRK channels by using whole-cell recordings can be additionally complicated by the combination of dendritic localization of GIRK channels and the low cell membrane input resistance. Raphe 5-HT neurons are suitable for studying GIRK channels since (*i*) their dendrites are relatively short and without extensive arborization and (*ii*) they are electrically compact, displaying high input resistance (≥1 GΩ) in whole-cell patch-clamp recordings in brainstem slice preparation. Importantly, we found that when GIRK channels are continuously activated by a concentration of 5-HT_1A_ receptor agonist which produces near-complete receptor stimulation, recorded whole-cell GIRK conductances display only slow and limited run-down, presumably caused by desensitization. This allows recordings of sufficient duration for a fairly accurate determination of the blocker's concentration-response relationship. Due to the run-down, the calculated EC_50_ and Hill slope values for the blockers are expectedly slightly under- and over-estimated, respectively. It is worth mentioning that when GIRK conductances in 5-HT neurons are activated by supramaximal concentrations of 5-HT_1A_ receptor agonists (e.g. bath application of 300 nM 5-CT or R(+)-8-OH-DPAT) or with the inclusion of non-hydrolyzable GTP analogue GTP-γ-S in the pipette solution (100 μM, instead of GTP) marked desensitization occurs ([6], our unpublished observation)

### Pharmacological properties of GIRK channels in 5-HT neurons only partially match those of GIRK1-GIRK2 heteromers

In the brain, GIRK1, GIRK2 and GIRK3 channel subunits are widely expressed, whereas the GIRK4 subunit is found at low levels. Although the precise subunit composition of GIRK channels in various neuronal subtypes and subcellular compartments is not entirely known (for recent reviews see [21,22]), there is evidence that GIRK1-GIRK2 heteromer is the predominant form of GIRK channels in neurons [23] and that GIRK2-containing channels mediate postsynaptic inhibition by G protein-coupled neurotransmitter receptors [24]. Neuronal GIRK channels may also be composed of different combinations of subunits, e.g. GIRK2A-GIRK2C [25], GIRK2-GIRK3 [26,27] and GIRK1-GIRK3 [28] and possibly of three different subunits [29]. All of these combinations are possible in 5-HT neurons, since they co-express GIRK1, GIRK2 and GIRK3 subunits [10].

Although the pharmacology of GIRK3-containing channels is still uncharacterized and subunit-selective GIRK blockers are presently lacking, some important conclusions regarding the molecular identity of GIRK channels in 5-HT neurons can be deduced from our findings. By comparing the effects of here characterized blockers with the published data on their activity at GIRK channels, it appears that 5-HT_1A_ receptor-activated inwardly rectifying K^+^ conductance only partially matches that of GIRK1-GIRK2 heteromers. Relatively good correspondence was found for tertiapin-Q which blocked 5-HT_1A_ receptor-activated GIRK conductance with an EC_50_ of ~34 nM, a value similar to those reported for blocking GIRK current in AtT-20 cells (102 nM; [20]) which express native mouse GIRK1 and GIRK2 subunits, and in HEK239 cells heterologously expressing mouse GIRK1 and GIRK2A or GIRK2C subunits (~76% and ~59% block by 100 nM, respectively; [30]). Similarly, the EC_50_ of SCH23390 (~2 μM) was in the range of those reported to block native mouse GIRK channels in AtT-20 cells (EC_50_ of 236 nM) and heterologously expressed human GIRK1-GIRK2 channels (EC_50_ of 7.8 μM) [18]. In contrast, our finding that U73343 and SCH28080 are poorly effective blockers of GIRK channels in 5-HT neurons significantly differs from previous studies that showed that U73343 blocks native GIRK channels in rat neocortical pyramidal neurons and heterologously expressed GIRK1-GIRK2 channels with EC_50_ of ~400 nM (~ 85% block by 10 μM) [19] and that SCH28080 blocks ~80% of native mouse GIRK channels in AtT-20 cells with an EC_50_ of ~200 nM [20]. Finally, Ba^2+^ blocked 5-HT_1A_ receptor-activated GIRK channels with the EC_50_ of 9.4 μM, a value close to that of 12 μM reported in rat CA3 pyramidal neurons, which express all GIRK subunits [31], but significantly lower than that reported for heterologously expressed mouse GIRK1-GIRK2 channels (EC_50_ of ~100 μM; [32]). Therefore, it seems unlikely that the 5-HT_1A_ receptor-coupled GIRK channels in dorsal raphe 5-HT neurons is a GIRK1-GIRK2 heteromer. In addition, GIRK2 homomer may also be excluded as a 5-HT_1A_ autoreceptor effector since SCH23390 blocks heterologously expressed human GIRK2 homomer with the EC_50_ of 83 μM [18], a value ~40 times higher than in 5-HT neurons.

The conclusion that GIRK1-GIRK2 heteromer and GIRK2 homomer are unlikely to be 5-HT_1A_ autoreceptor-coupled channels in 5-HT neurons is further supported by expression studies that found a low level of GIRK2 mRNA and protein in rat dorsal raphe [9,10] or failed to detect GIRK2 mRNA [7] and protein [8] at all. In addition, a recent functional study in mice *in vivo* [33], showed that genetic deletion of GIRK2 does not abolish 5-HT_1A_ receptor-mediated suppression of 5-HT neuron firing, but only results in a limited rightward shift of dose-response curves for 8-OH-DPAT, a 5-HT_1A_ receptor agonist, and citalopram, a selective serotonin reuptake inhibitor (SSRI).

Although further studies are clearly needed to define the molecular identity of GIRK channels in 5-HT neurons, their distinct pharmacological properties might provide the foundation for the development of 5-HT neuron- selective/preferential GIRK blockers. Such drugs would have a potentially important therapeutic role since they should increase 5-HT neuron activity without causing serious side effects by the nonselective block of GIRK channels, in particular of neuronal GIRK1-GIRK2 and cardiac GIRK1-GIRK4 heteromers. In fact, the delay in the therapeutic effect of SSRIs, the most commonly prescribed antidepressants, has in part been ascribed to the drug-induced increase in raphe 5-HT level, which decreases 5-HT neuron activity by activating 5-HT_1A_ autoreceptors [34]. Thus, 5-HT neuron-specific GIRK blockers in association with SSRIs, may afford a valuable therapeutic strategy to hasten the antidepressant effects.

### Tertiapin-Q-resistant conductance

The finding that ~16% of total 5-HT_1A_ autoreceptor-activated inwardly rectifying K^+^ conductance is tertiapin-Q-resistant was unexpected. The lack of a complete block is unlikely to be caused to impurities in tertiapin-Q since identical results were observed with several different batches of tertiapin-Q obtained from two different commercial sources. We cannot exclude that part of the 5-HT_1A_ autoreceptor-activated tertiapin-Q-resistant conductance is mediated by ion channels other than GIRK, but this seems unlikely since 100 μM Ba^2+^ and 30 μM SCH23390 produced full block. It is conceivable that a subpopulation of GIRK channels in 5-HT neurons has a distinctive GIRK subunit composition that results in tertiapin-Q-resistance. In fact, differential sensitivity of rat GIRK subunits to tertiapin-Q has been demonstrated by Ramu *et al*. [35], who showed that high tertiapin-Q affinity of cardiac GIRK1-GIRK4 heteromers (K_d_ ≈ 13 nM, [16]) results from interaction of tertiapin-Q with GIRK4 subunit (K_d_ ≈ 2 nM), while GIRK1 is relatively insensitive (K_d_ ≈ 20 μM). Therefore, it is reasonable to assume that the tertiapin-Q-resistant conductance in 5-HT neurons is mediated by a GIRK1-containing channels. Other subunits besides GIRK1 may contribute to differential sensitivity to tertiapin-Q. As example, Walsh [20] reported that tertiapin-Q blocks native mouse GIRK1-GIRK4 and GIRK1-GIRK2 channels with a two orders of magnitude different potency (EC_50_ of 1.4 and 102 nM, respectively). Finally, tertiapin-Q-resistant channels may result from promiscuous GIRK1-IRK heteromerization, which has been demonstrated feasible by Ishihara *et al*.[36].

## Conclusions

In conclusion, tertiapin-Q, SCH23390 and Ba^2+^ are useful tools for studying GIRK function in 5-HT neurons, albeit with specific limitations since tertiapin-Q does not produce full block whereas SCH23390 and Ba^2+^ do not warrant selectivity of action on GIRK channels. Importantly, our data show that 5-HT_1A_ autoreceptor-coupled GIRK channels in 5-HT neurons have specific pharmacological properties, which may provide foundation for the development of valuable new therapeutic agents.
